# Persistent Immune Thrombocytopenia Resistant to Immunosuppressive Therapy: What Is the Way Forward?

**DOI:** 10.7759/cureus.12377

**Published:** 2020-12-30

**Authors:** Muhammad Hafiz Kamarul Bahrin, Harini Vijayenthiran, Laura Stimson, Humayun Ahmad

**Affiliations:** 1 Internal Medicine, United Lincolnshire Hospitals NHS Trust, Boston, GBR; 2 Haematology, University Hospital of Derby and Burton NHS Trust, Burton-On-Trent, GBR

**Keywords:** immune thrombocytopenia purpura, haemorrhagic bullae, romiplostim

## Abstract

Immune thrombocytopenia purpura (ITP) involves autoimmune induced platelet destruction and decreased platelet production in part due to autoantibody destruction mechanisms. Most autoantibodies involved in its pathogenesis invoke autoreactive T cells and cytokine imbalance, and most drug therapies target these mechanisms. We describe a man in his late 40s, with a medical history of ITP, who presented with blood blisters on his mucosal surfaces and bruises on all four limbs with petechial rashes. He subsequently developed epistaxis and hemoptysis. In the recent past, he had been camping in Malta and felt feverish and nauseous on return. This was his first relapse of the disease in six years, and was unresponsive to prednisolone, IV immunoglobulins, and methylprednisolone, subsequently requiring romiplostim to recover platelet counts and reduce bleeding. When investigating the underlying causes of thrombocytopenia, aspects of virology and rickettsial serology were positive, requiring precautionary measures with long-term maintenance immunosuppression to prevent reactivation of infection.

## Introduction

Immune thrombocytopenia purpura (ITP) is an uncommon hematological disorder characterized by isolated thrombocytopenia in the absence of systemic illness. It occurs in about 1-2 cases per 100,000 population worldwide with a mean age of presentation of 50 years. Whilst it may happen in young children, the prognosis is generally good, and they often achieve complete remission [1]. Adults tend to develop a more chronic pattern of the disease and often require medications. The prognosis for older adults is generally poor if they show no response to the initial therapy [2]. It was postulated that in ITP, autoantibodies opsonizes on the platelet membrane resulting in reduced platelet survival by the reticuloendothelial system [3]. 

The clinical presentation of ITP varies and is usually related to the thrombocytopenic state, i.e., when platelet level falls below 150 x 10^9^/L. Symptoms of petechiae, gingival bleeding, and epistaxis can occur when platelets decrease below 50 x 10^9^/L; however, emergencies such as gastrointestinal bleeds, intracranial hemorrhages can also occur and should be examined for [4]. There are several medications available on the market to treat ITP, and they suppress the autoantibodies-invoked autoreactive T cells and cytokine imbalance, which are known to play roles in its pathogenesis.

This case history was an example of a dramatic presentation of severe immune thrombocytopenia, resistant to treatment in a young patient requiring an increased potency of immunosuppression. It also highlights the complications, workup, and precautions needed with such medications, as well as a revision of context-dependent causal factors of thrombocytopenia.

## Case presentation

**Figure 1:**
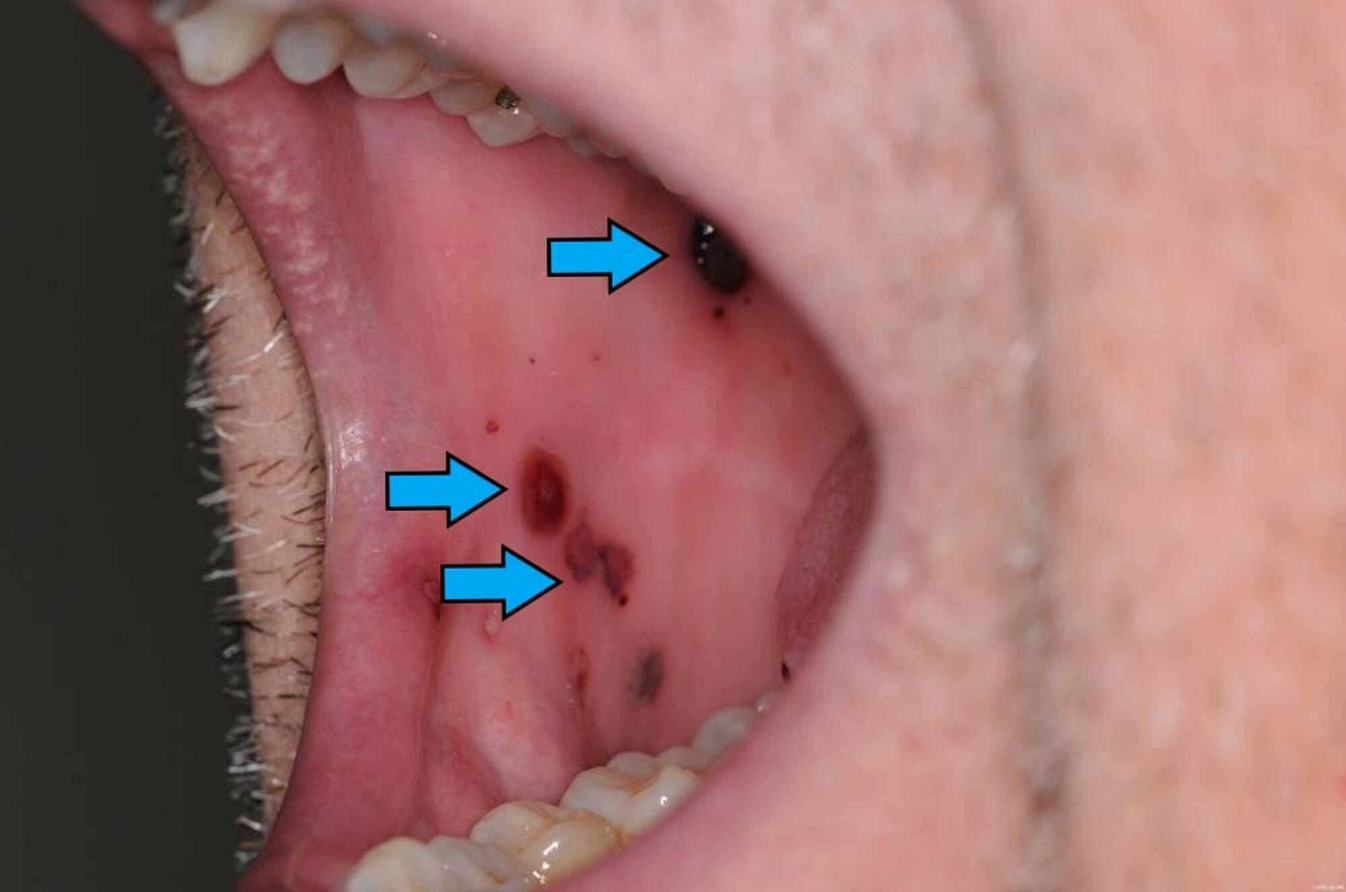
The initial presentation of our patient's refractory ITP with hemorrhagic oral mucosal bullae ITP - immune thrombocytopenia purpura

We report on a male patient in his late 40’s, previously diagnosed with immune thrombocytopenia presented with painless blood blisters in mouth and lips (Figure 1), which progressed to nose bleeds and intermittent hemoptysis. Since diagnosis in 2002, he has been in remission. Two weeks prior to this relapse, he had been swimming and camping in Malta, where he felt feverish with prolonged night sweats and diarrheal illness. There was no recent change in medication, no recent blood transfusions, or hospital admissions. More specifically, there was no recorded history of liver disease or other autoimmune conditions. He was previously in the army touring in Afghanistan and therefore received a large range of atypical vaccinations seven years ago. 

**Figure 2:**
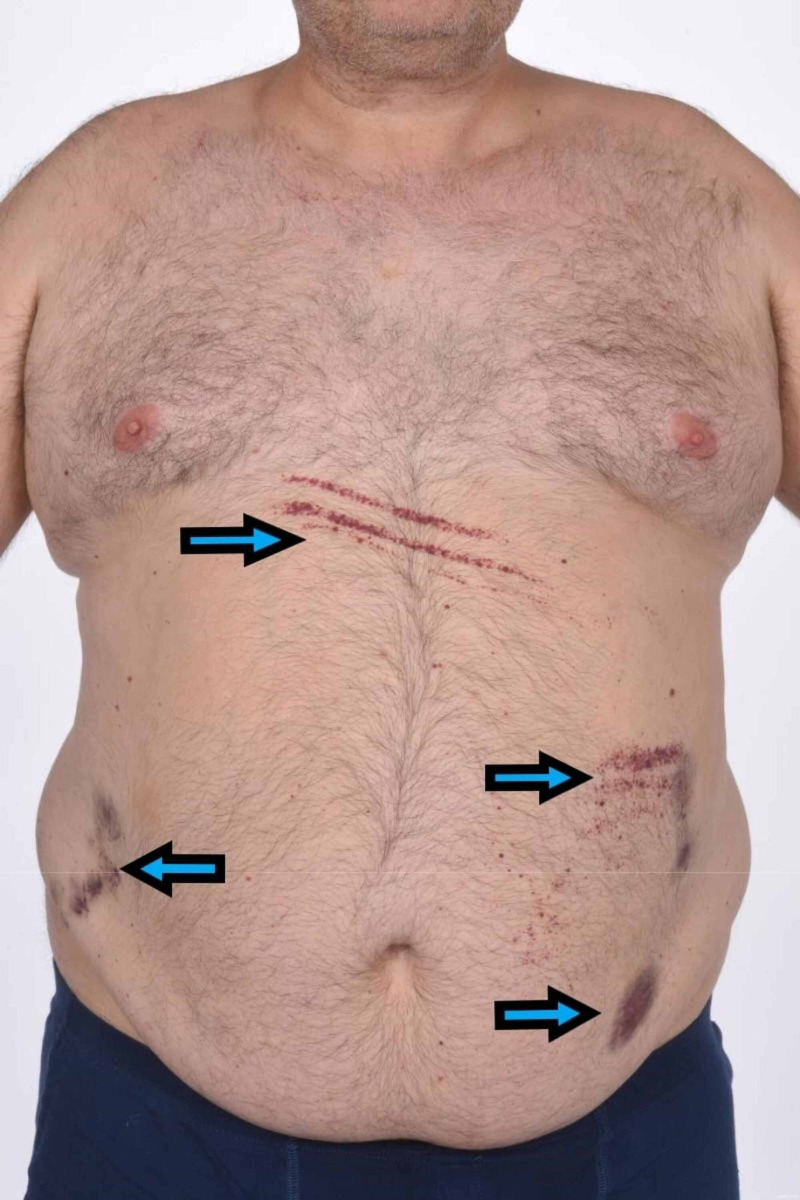
Multiple contact-induced petechiae and ecchymoses on patient's abdomen

On examination, there were hemorrhagic blisters on mucosal mouth surfaces, bruises on the trunk (Figure 2) and medial aspects of all four limbs, accompanied by bilateral petechial ankle rashes. In particular, there was no lymphadenopathy or history of weight loss.

Investigation

The patient's platelet counts were initially 2 (150-410) x 10^9^/L. The purpose of the following workup was to rule out life-threatening causes of low platelets such as sepsis, thrombotic thrombocytopenic purpura (TTP), or underlying neoplasm.

Since this relapse was aggressive in presentation and a large number of years had passed since the previous episode, the trigger factor cause was sought for the thrombocytopenia:

1) Renal and liver function were satisfactory, suggesting that differentials of hemolytic uremia syndrome or chronic liver disease are unlikely.

2) Peripheral blood smear was examined for clumping and morphology of all three cell lines. There were no appearance of abnormal cells such as schistocytes and laboratory evidence of hemolysis with hemoglobin, lactate dehydrogenase, direct antiglobulin screen, and reticulocytes and bilirubin were normal.

3) ITP can be associated with a myeloid or lymphoproliferative disorder; however, his bone marrow biopsy showed all stages of myeloid maturation with no excess of lymphocytes, blast, or plasma cells. A good number of megakaryocytes present, indicating that there was no bone marrow failure and therefore correlates more with the peripheral consumption of platelets.

4) Bronchoscopy was sought as the patient suffered recurrent hemoptysis that was unamenable to antibiotics. No neoplastic lesions were seen but petechial hemorrhages on pleura, which could coincide with platelet consumption or vasculitic changes.

5) Secondary viral screen for hepatitis was positive for hepatitis B core antibody and hepatitis B surface antibody 1000miu/ml, indicating a previous exposure and natural immunity.

6) Tick-borne virus screen was positive for immunoglobulin G (IgG) tick-borne rickettsial spotted fever with titer 1:64.

7) Investigating vasculitic causes of hemoptysis and ITP in a young man returned a positive connective tissue disease screen but negative antineutrophil cytoplasmic antibody (ANCA), C3, C4, lupus anticoagulant.

8) Infective causes such as atypical pneumonia and H. pylori bacteria were also sought on sputum, stool tests and urine cultures, which were also negative.

Management

The goal of the treatment was to increase platelet count and to achieve hemostasis. Initially, he was treated with oral glucocorticoid in the form of prednisolone 30mg once daily for two days; however, as this was an aggressive relapse, we decided to commence intravenous (IV) immunoglobulins at 1 mg/kg over the next two days. Despite this, the platelet count failed to improve. Subsequently, five days of IV methylprednisolone 1 g/day were given. Lack of response to the standard first and second-line therapy was evident when the platelet level showed no improvement at all after a week, and the patient complained of recurring hemoptysis. 

At this point, we diagnosed the patient as refractory ITP, and advice on further management was sought from the regional hematology center. Following this, the patient was started on oral azathioprine 1 mg/kg once daily in combination with IV romiplostim, a fusion protein analog of thrombopoietin, at a dose of 1 microgram/kg once weekly. 

The platelets rose to 360(150-410) x 10^9^/L over the course of a week.

Supportive platelet transfusions were given before bronchoscopy and bone marrow biopsy with an aim to maintain platelets count above 50 x 10^9^/L. Platelet transfusions were not employed with the aim of treating platelet count as they were peripherally broken down.

Antibiotics were given prophylactically to treat coinciding atypical pneumonia due to the presence of recurrent hemoptysis, fevers on presentation.

Repeated doses of tranexamic acid 1 g three times daily for the hemoptysis and relevant prophylaxis in the form of graduated compression stockings were prescribed to prevent thrombosis.

Once the hemoptysis resolved and platelet numbers stabilized, maintenance of remission was achieved through long term immunosuppression with azathioprine 1 mg/kg daily with a tapering course of prednisolone to bridge the therapeutic treatment window.

Discussion with Infectious diseases team regarding treating rickettsial positive serology in a patient with fevers and long-term immunosuppression was needed and prophylactic course of doxycycline 100 mg once daily was prescribed for seven days in case of an early rickettsial spotted viral infection. The role of his rickettsial IgG seropositive status during this presentation on his acute exacerbation of ITP is inconclusive; however, they could be related to each other. Advice from the hepatology team was also sought in terms of reactivation of hepatitis B in the context of azathioprine suppression and whether prophylactic viral treatment was needed. The decision made was to maintain his current immunosuppressive therapy while having regular outpatient hepatology reviews for a watch-and-wait intent.

On subsequent outpatient visit six months after the acute admission, our patient's platelet count remained above 350 x 10^9^/L. He did not experience significant side effects with the tapering steroid dose and has been left on azathioprine maintenance dose.

## Discussion

This patient already had a background diagnosis of immune thrombocytopenia that was previously responding well to a short course of steroids achieving remission. Thrombocytopenia as a singular deficiency can be dangerous as there is a large risk of internal bleeding that cannot always be accounted for. This patient's presentation was aggressive with epistaxis, hemoptysis, and hemorrhagic bullae. The nonresponse to first-line treatment with steroids and, therefore, the need for stronger immunosuppression makes this case challenging in the background of previous hepatitis and rickettsial disease exposure.

Corticosteroids are generally used as first-line treatment. Their mechanisms of action target cytokine-induced inflammation [5]. Following the patient’s refractory response to methylprednisolone, a synthetic corticosteroid achieving higher bioavailability than prednisolone, with properties of immunomodulation, was utilized. By altering gene expression and stopping cytokine production, these drugs reduce the number of circulating lymphocytes. The overall impact of the above is in a reduced recruitment of B lymphocytes and hence, their subsequent production of auto-antibodies against platelets [6].

Alongside steroids, IV immunoglobulins are given to provide non-competitive inhibition on receptors of the phagocytic cells within the reticuloendothelial system in an attempt to reduce peripheral platelet destruction [7]. Authors are aware that some centers utilize monoclonal antibody therapy such as rituximab as the second-line therapy in treating refractory ITP, either as a monotherapy or in combination with corticosteroid drug. However, in our case, the patient was not given this medication for two reasons. Firstly, it is a B cell depleting therapy, hence given his previous history of hepatitis B, it would put him at risk of disease reactivation. Secondly, there is no strong evidence for the use of rituximab in treating refractory ITP according to the UK national guideline (published by the National Institute of Health and Care Excellence, otherwise known as NICE) - the recommendation comes mostly from observational studies with no comparator arm and the randomized controlled trials (RCT) on its use has adverse limitations such as small participants cohort [8]**.**

Romiplostim, a fusion protein, is utilized in patients who have had poor outcomes with corticosteroids and immunoglobulins and acts by increasing platelet production by binding to the thrombopoietin receptor in the bone marrow. When discontinuing this drug, there are cautions surrounding developing reticulin fiber deposition in the bone marrow and temporary worsening bleeding. Therefore close monitoring of the patient for bleeding exacerbations is warranted [9]. A UK-based RCT by Cooper et al. demonstrated remarkable clinical efficacy of romiplostim use in both non-splenectomized and splenectomized patients with refractory ITP, i.e., platelet count of at least 50 x 10^9^/L was maintained for 15.2 and 12.3 weeks, respectively, as compared to 1.3 and 0.2 weeks, respectively, in placebo groups. A similar result is demonstrated by a Japanese RCT by Shirasugi et al., according to which the mean change from the baseline platelet count was significantly greater in the romiplostim arm versus the placebo arm (110 vs. 2 × 10^9^/L) with a p-value of 0.0003 [10].

B Cell lymphocytes, which are recruited less in steroid therapy, are a type of white cell that is a component of the adaptive immune system. When commencing an immunosuppressive treatment, infections such as blood-borne viruses and tuberculosis (TB) that can escape immune control need to be checked for as they have an increased chance of replicating and reactivating.

Splenectomy is generally reserved for chronic ITP, which shows no response to medical treatments. In view of the advent of medical alternatives nowadays, its use has generally declined [11].

The stronger the immunosuppression, the higher the risk of resetting of the immune system whilst, following the withdrawal of treatment, can also provoke a reactivation, which could be fatal. This is particularly the case in those with positive hepatitis B surface antigen (HBsAg) status. However, those with positive hepatitis B core antibody (anti-HBc) despite negative HBsAg status (indicating past infection) could still be at risk as the latent virus may reactivate following immunosuppression [12].

As this patient falls under the latter category, prophylaxis with lamivudine would need to be given with B cell depleting therapies such as rituximab. Since the treatment team proposed azathioprine and prednisolone as maintenance, which is not specifically designed to deplete B cells but to reduce its recruitment and activation by cytokines, no prophylaxis for hepatitis B was needed.

During this patient’s viral and tick-borne illness screen, he was positive for rickettsial IgG serology. This could suggest exposure to antigens from vaccination at some point but also early infection. With a low titer and negative immunoglobulin M (IgM), an active infection with rickettsial parasite is unlikely. However, given his symptoms of night sweats and fevers as an inpatient, it was decided to give him a course of doxycycline to treat this prophylactically. To the authors' knowledge, a causative relationship between acute rickettsial infection and primary ITP has been demonstrated in only one case report so far. This was explained through 'molecular mimicry' whereby the same antibodies produced targeting the rickettsial parasites also show cross-reactivity with platelet glycoproteins, leading to peripheral platelet consumption [13]. As for acute rickettsial infection leading to exacerbation of an underlying stable ITP, authors are not aware of the evidence for this, and further studies may be needed. 

## Conclusions

The case above describes a severe ITP unresponsive to the standard first and second-line therapy. In the first instance, it is important to take into account the immunosuppression ladder. General drug classes can include corticosteroids, Janus kinase inhibitors, which focus on lymphocytes, calcineurin inhibitors, biological antagonists against certain cell markers, and monoclonal antibodies. Corticosteroids are utilized as the first-line therapy in ITP due to their effects in altering the transcription of genes in leucocytes and reducing cytokines, resulting in a simple immunomodulation effect without significant immunosuppression. The step up from this is usually IV Immunoglobulins, which act via noncompetitive inhibition receptors on phagocytic cells within the reticuloendothelial system. In patients who remain unresponsive to therapies discussed above, purine analogs combined with a thrombopoietin analog protein such as romiplostim could be used. Once medical treatments are exhausted, splenectomy could be considered.
